# From lab to life: challenges and perspectives of fNIRS for haemodynamic-based neurofeedback in real-world environments

**DOI:** 10.1098/rstb.2023.0087

**Published:** 2024-10-21

**Authors:** Franziska Klein, Simon H. Kohl, Michael Lührs, David M. A. Mehler, Bettina Sorger

**Affiliations:** ^1^ Biomedical Devices and Systems Group, R&D Division Health, OFFIS—Institute for Information Technology, Oldenburg, Germany; ^2^ Department of Psychiatry, Psychotherapy and Psychosomatics, Medical School, RWTH Aachen University, Aachen, Germany; ^3^ JARA-Institute Molecular Neuroscience and Neuroimaging (INM-11), Forschungszentrum Jülich, Jülich, Germany; ^4^ Child Neuropsychology Section, Department of Child and Adolescent Psychiatry, Psychosomatics and Psychotherapy, Faculty of Medicine, RWTH Aachen University, Aachen, Germany; ^5^ Department of Cognitive Neuroscience, Faculty of Psychology and Neuroscience, Maastricht University, Maastricht, The Netherlands; ^6^ Brain Innovation B.V., Research Department, Maastricht, The Netherlands; ^7^ Institute of Translational Psychiatry, Medical Faculty, University of Münster, Münster, Germany; ^8^ Cardiff University Brain Research Imaging Centre (CUBRIC), School of Psychology, Cardiff University, Cardiff, UK

**Keywords:** brain haemodynamics, fNIRS, optical imaging, real-time/online analysis, neurofeedback, neurotherapy

## Abstract

Neurofeedback allows individuals to monitor and self-regulate their brain activity, potentially improving human brain function. Beyond the traditional electrophysiological approach using primarily electroencephalography, brain haemodynamics measured with functional magnetic resonance imaging (fMRI) and more recently, functional near-infrared spectroscopy (fNIRS) have been used (haemodynamic-based neurofeedback), particularly to improve the spatial specificity of neurofeedback. Over recent years, especially fNIRS has attracted great attention because it offers several advantages over fMRI such as increased user accessibility, cost-effectiveness and mobility—the latter being the most distinct feature of fNIRS. The next logical step would be to transfer haemodynamic-based neurofeedback protocols that have already been proven and validated by fMRI to mobile fNIRS. However, this undertaking is not always easy, especially since fNIRS novices may miss important fNIRS-specific methodological challenges. This review is aimed at researchers from different fields who seek to exploit the unique capabilities of fNIRS for neurofeedback. It carefully addresses fNIRS-specific challenges and offers suggestions for possible solutions. If the challenges raised are addressed and further developed, fNIRS could emerge as a useful neurofeedback technique with its own unique application potential—the targeted training of brain activity in real-world environments, thereby significantly expanding the scope and scalability of haemodynamic-based neurofeedback applications.

This article is part of the theme issue ‘Neurofeedback: new territories and neurocognitive mechanisms of endogenous neuromodulation’.

## 1. Functional near-infrared spectroscopy neurofeedback: a promising but challenging neuromodulation approach

**Figure 1.**
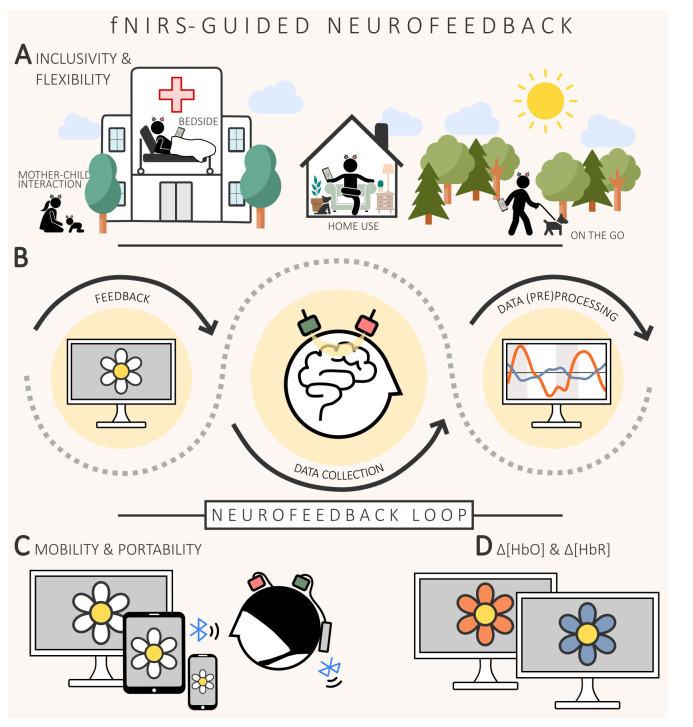
Illustration of the advantages and basic principles of fNIRS neurofeedback. (*a*) Emphasizes the inclusivity and flexibility of fNIRS and highlights its potential for real-world applications such as bedside use, home (neuro)therapy and mobile scenarios. It also shows the adaptability of fNIRS to different body positions and its applicability in different populations, including children, and in unique future configurations such as hyperscanning neurofeedback (‘hyperfeedback’) scenarios with mother–child interactions (cf. §4). (*b*) Illustrates the neurofeedback loop: brain activity is measured by fNIRS, (pre)processed and then used to generate a (visual, auditory or tactile) feedback representation (illustrated here as visual feedback in form of a flower representation). (*c*) Highlights the advances in mobile and wireless fNIRS technology that further improve its applicability in real-world environments. (*d*) Furthermore, unlike fMRI, fNIRS offers the choice between Δ[HbO] (red flower) and Δ[HbR] (blue flower) as neurofeedback information, potentially enabling more personalized neurofeedback approaches.

Functional neuroimaging exploiting brain haemodynamics has advanced rapidly over the past three decades, allowing neuroscientists to delve deeper into the understanding of the human brain than ever before possible [1]. A fascinating development in this context is the provision of individuals with haemodynamic (i.e. functional near-infrared spectroscopy (fNIRS)- or functional magnetic resonance imaging (fMRI)-based) neurofeedback—a representation of (an aspect of) the haemodynamic brain response—that indirectly allows monitoring and modification of ongoing brain activity [2]. The main purpose of this approach is for individuals to learn to self-regulate their own brain activity in a desired direction by interpreting and responding appropriately to the neurofeedback information (cf. figure 1*b*—neurofeedback loop). Possible areas of application range from ‘neurotherapy’ of psychiatric and neurological diseases to ‘neuroenhancement’, that is, improving brain function in healthy populations. The specific generation and type of neurofeedback information provided may vary. Comprehensive overviews of the haemodynamic-neurofeedback methodology can be found in several review articles [2–5].

The beginnings of neurofeedback as a neuromodulation technique go back to the use of electroencephalography (EEG), in which electrical potentials of neurons in the brain are directly measured [6,7]. In most cases, EEG neurofeedback results in the modulation of spontaneous brain rhythms, which are characterized primarily by their frequency [8,9]. As an alternative to using electrophysiological signals, the haemodynamic approach gained increasing attention in the field of neurofeedback in the early 2000s. FMRI, which is based on the blood oxygen level-dependent (BOLD) effect, emerged as a popular choice due to its significantly higher spatial resolution compared to EEG. The innovation of fMRI made it possible to provide neurofeedback information from a more locally specific brain region of interest (ROI) [10]. Since its introduction, research has primarily focused on further developing the fMRI-neurofeedback method, which ultimately led to this technique now being used in first clinical trials with the aim of demonstrating its effectiveness in patient populations [11–15]. However, the inherent limitations of fMRI, particularly its significant cost, overall complexity and immobility, represent significant barriers to widespread application of fMRI neurofeedback and reduce the prospects for its future integration into routine clinical practice.

Parallel to these developments, fNIRS emerged as an alternative non-invasive haemodynamic brain-imaging technique with the first description of an fNIRS device published in 1993 [16]. The functional principle of fNIRS is based on NIRS developed by Jöbsis in the 1970s [17]. FNIRS uses two or more wavelengths in the NIR range and sends them into the scalp, skull and finally the brain to measure changes in the concentrations of certain chromophores, namely oxygenated ( Δ[HbO] ) and deoxygenated ( Δ[HbR] ) haemoglobin in the small blood vessels within the superficial cortical layers [18–20]. These concentration changes are derived from the amount of light backscattered to one or more detector optodes typically located at a distance of about 3 cm from the source optode [18]. FNIRS has evolved significantly, especially in recent years, and has become a widely used research tool in basic and various applied neuroscience disciplines [16,21–25]. Generally, fNIRS offers several unique advantages over fMRI that have led to its increased application. Foremost, fNIRS allows measuring haemodynamic brain signals in real-world environments [26] and during natural behaviour (cf. figure 1*a*) [27]. This is possible due to its portability/mobility (cf. figure 1*c*), simple operating principles and lower sensitivity to motion artefacts compared to other techniques such as EEG and fMRI.

The specific potential of fNIRS for haemodynamic-based neurofeedback applications is based on a variety of further advantages (cf. table 1 for a complete overview). Its general suitability for clinical applications is based on its complete safety, ease of application, motion tolerance, environmental flexibility, cost-effectiveness, user-friendliness and comfort—allowing frequent, long-term/continuous measurements of even two different brain-signal types ( Δ[HbO] and Δ[HbR] . figure 1*d*). However, like any other functional neuroimaging method, fNIRS has several methodological challenges, particularly its limitations in (full) brain coverage and spatial resolution, as well as its general susceptibility to various noise sources (cf. table 1). It is important for researchers and clinicians to recognize and carefully consider these challenges when designing experiments and developing clinical applications. However, with the rapid advancement of technology, it is likely that several of the stated challenges can be mitigated (cf. §§3 and 4), potentially further increasing the usefulness of fNIRS in general and for neurofeedback applications in particular.

**Table 1.** Advantages and challenges of fNIRS and resulting implications for neurofeedback applications (and possible countermeasures). HD-DOT, high-density diffuse optical tomography.

	description	resulting implication for neurofeedback (and countermeasure)
*advantages*		
compactness of the device	fNIRS equipment is portable and often mobile	possibility to perform neurofeedback in real-world environments (e.g., at home or bedside) and in low-resource countries as well as while performing natural tasks
minimal discomfort	fNIRS does not produce loud noises or require patients to lie still inside a confined space (as fMRI)—meaning reduced stress, anxiety and discomfort	increased user tolerability and possibility to involve sensitive/vulnerable populations (e.g. children, psychiatric and elderly patients)
low-tech nature	in comparison to (high-tech) fMRI, fNIRS is relatively low-tech, translating to considerably lower purchase, operating and maintenance costs	increased affordability/accessibility for fNIRS neurofeedback
body-posture independence	fNIRS is functional regardless of the individual’s body position (i.e., sitting, lying and standing)	increased flexibility/applicability for patients in different clinical conditions (e.g., bedside, wheelchair)
motion tolerance	fNIRS is less sensitive to motion artefacts compared with fMRI and EEG	the robustness to motion artefacts is particularly advantageous for clinical neurofeedback applications
technical compatibility with paramagnetic equipment	fNIRS is compatible with paramagnetic (incl. medical) equipment or other techniques for recording or interfering with brain activity (e.g. EEG, TMS, fMRI)	enables simultaneous measurements of electrophysiological and haemodynamic brain activation to provide multi-modal (neuro)feedback which might offer novel, more effective and efficient neurofeedback applications
ease of application	setting-up and calibrating fNIRS equipment is remarkably quick and straightforward; there is no need for gel application and scalp scrubbing (as in EEG), which simplifies the preparation process	fNIRS neurofeedback might be applicable by non-experts; ideally, patients could operate it independently or with the assistance of a family member; this would allow for more frequent but shorter neurofeedback sessions—a procedure being much more suited in the clinical context
full safety	fNIRS is a non-invasive technique that uses low-energy near-infrared light without the need for ionizing radiation, radio frequency pulses or strong magnetic fields	the full safety makes it an ideal neurofeedback method for repeated and long-term use even in vulnerable populations (e.g. patients and children)
availability of both *HbO* and *HbR* changes	fNIRS quantifies changes in both oxygenated ( Δ[HbO] ) and deoxygenated ( Δ[HbR] ) haemoglobin related to brain activity	the possibility to alternatively use Δ[HbO] or Δ[HbR] enables more personalized neurofeedback approaches; when combining both Δ[HbO] and Δ[HbR] for neurofeedback, the robustness and validity of the provided neurofeedback information can be increased
relatively high single-trial reliability	like fMRI, fNIRS has higher single-trial reliability compared to EEG and allows researchers to obtain more consistent results across repeated trials [28]	higher single-trial reliability results in increased effectiveness of neurofeedback learning
*challenges*		
indirectly measuring brain activity	like fMRI, fNIRS relies on changes in blood (de)oxygenation that are associated with changes in brain activity	limited effectiveness: modulation of neural activity via its brain haemodynamics may result in reduced precision and effectiveness; while this is a given fact, the simultaneous use of electric and haemodynamic methods can potentially improve the effectiveness of neurofeedback interventions
lack of anatomicalinformation	unlike fMRI, fNIRS does not provide individual anatomical information	limited spatial specificity: given the significant inter-individual variability in brain size and structure, this hinders the ability to precisely target specific brain regions for neurofeedback; addressable by applying advanced methods such as neuronavigation, combinatorial use with fMRI, probabilistic approaches, etc.
limited spatial resolution	fNIRS typically provides a spatial resolution of approximately 1 cm ${,}^{3}$ [27] which is significantly lower than that achieved with fMRI	limited spatial specificity: desired changes in brain activation presented at a fine spatial scale may not be specifically altered by fNIRS neurofeedback; addressable to some degree by using advanced methods such as high-density fNIRS or HD-DOT [29–31]
limited depth pervasion	fNIRS mainly captures superficial cortical activity due to its limited penetration depth of approximately 1.5 cm [27]	limited applicability: the fNIRS neurofeedback approach is not suitable for targeting deeper cortical and subcortical regions; partially addressable through indirect training of deeper brain regions via functional connectivity
signal-quality variability across individuals	physical features of individual participants (e.g. skull thickness, skin pigmentation, hair pigmentation, texture and colour) vary significantly and may negatively affect the quality of the fNIRS signal through increased light scattering/absorption [32]	limited applicability: the fNIRS neurofeedback approach may not work equally well for every individual and may not be suitable at all for a portion of the general population (i.e. ‘good’ versus ‘poor’ responders); while most physical characteristics are invariant, a better understanding of the relationship between physical features and the quality of the fNIRS signal would be beneficial, e.g. in predicting neurofeedback performance
limited brain coverage	typically, the number of affordable/available optodes is limited and only covers part of the brain or even the hemisphere	limited applicability: simultaneous training of highly distributed brain regions/large brain networks may not be possible; reduced comfort: increasing the number of optodes is associated with increased burden/lower compliance on the participants’ side; in principle addressable by developing high-density systems which are at the same time still comfortable
susceptibility to artefacts	like fMRI, fNIRS is highly susceptible to both task- and non-task evoked (extra)cerebral systemic physiological artefacts/noise (e.g. caused by changes in blood pressure and heart rate) 33]	limited effectiveness: provided fNIRS neurofeedback information may not be reliable/robust enough to effectively learn modulating the haemodynamic response; addressable by advanced artefact control in real-time (i.e. individualized filtering and short-channel regression) and carefully considered study designs (proper timing of modulation and rest periods)
limited temporal resolution	while the sampling rate of fNIRS is considerably higher than in fMRI, the temporal resolution is still limited by the biological constraint of the delay of the haemodynamic response	limited effectiveness: the (desired) concurrency of applying a specific cognitive modulation strategy and the resulting neurofeedback information is limited—this could slow down neurofeedback learning; in addition, the limited temporal resolution results is a relatively low number of (neurofeedback) trials; partially addressable by implementing proper study design and appropriate task instruction)
possible discomfort wearing fNIRS cap/headband	longer wear times of fNIRS cap/headband can become uncomfortable due to applied pressure of optodes (ensuring sufficient optode-skin contact) [34]	limited applicability: complaints can lead to the fNIRS neurofeedback training being discontinued/rejected outright; limited effectiveness: fNIRS neurofeedback signal might be affected by pain/discomfort-related brain activity or increase of systematic physiology [33,35,36]; addressable to some degree by careful optode placement and collaboration with fNIRS manufacturers to develop more patient-friendly fNIRS equipment

This review is aimed at both fNIRS novices and experienced users from various areas of basic and applied neuroscience who would like to make optimal use of the capabilities of fNIRS neurofeedback in future studies. After familiarizing the reader with the advantages and challenges of fNIRS as functional neuroimaging method as well as their implications (and countermeasures) for neurofeedback applications (cf. table 1), we will discuss ways to translate promising fMRI neurofeedback research into different cognitive domains on mobile, lower-cost and easier-to-implement fNIRS neurofeedback protocols. We also provide a brief overview of existing empirical work on fNIRS neurofeedback (cf. §2). Furthermore, a substantial part of this review is dedicated to presenting the various methodological advances that are being developed to address some of the challenges previously discussed. These advances are particularly important because they have the potential to improve the implementation and dissemination of fNIRS neurofeedback (cf. §3). Finally, we will present our vision for future fNIRS neurofeedback methodology and application (cf. §4).

## 2. Translating fMRI neurofeedback to fNIRS: what is possible?

The aim of this section is to discuss a series of selected clinical fMRI neurofeedback studies that we believe have great potential for adaptation to fNIRS neurofeedback protocols. At the same time, we provide a brief overview of recent fNIRS neurofeedback studies (for a more comprehensive overview see [3,10]). For general considerations regarding the design of neurofeedback interventions, we refer to the existing guiding literature [37–39].

When transferring fMRI neurofeedback protocols to fNIRS, it is important to understand the relationship of the fMRI BOLD signal and the two distinct signal types Δ[HbO] and Δ[HbR] obtained with fNIRS. Note that the time courses of Δ[HbO] and Δ[HbR] are usually highly negatively correlated, with Δ[HbO] increasing and Δ[HbR] decreasing when neurons within a brain region get more activated. This means that an fMRI BOLD upregulation protocol could be translated into either training to increase Δ[HbO] or to decrease Δ[HbR] (cf. figure 1*b–d*). Another crucially important consideration when translating fMRI to fNIRS protocols is that target ROIs must be compatible with the depth penetration and spatial resolution of fNIRS. As explained above, fNIRS is only sensitive to superficial cortical areas and the spatial resolution of standard fNIRS is significantly lower than in fMRI.

Most fMRI neurofeedback studies to date have applied univariate analysis approaches (e.g. training the mean brain activation within a single ROI) [1,5,40], although multivariate approaches that analyse multiple variables simultaneously and can therefore detect more complex patterns of brain activity are becoming increasingly important [41]. Given the higher spatial resolution required for multivariate approaches [42], univariate methods are generally more suitable for fNIRS neurofeedback protocols, as reflected in the majority of existing fNIRS studies.

Taking these factors into account, certain brain regions and their associated functions appear to be particularly well suited for translating neurofeedback protocols from fMRI to fNIRS. We identified four groups of brain functions, for some of which empirical fNIRS neurofeedback literature is already available.

### (a) Motor system function

In the area of motor function, motor imagery techniques (i.e., the mental imagination of a movement without actually executing it) are often used in fMRI neurofeedback studies. Since motor imagery involves motor networks that largely overlap with those activated during real movement execution (see for review [43,44]), it can be used as a training technique for motor rehabilitation [45], as well as for active forms of neuromodulation, including neurofeedback (see for review [46,47]). FMRI neurofeedback studies have used motor imagery to, for example, induce neuroplasticity in the motor system, reduce motor deficits and improve motor function in neurological diseases such as Parkinson’s [48–50] and Huntington’s disease [51], as well as ischaemic stroke [46,52]. A commonly used protocol involves the upregulation of the BOLD signal in ROIs such as the supplementary motor area (SMA) and premotor cortex (PMC) using kinaesthetic motor imagery (see for review [44,46,47]). This approach has shown substantial signal modulation in both healthy individuals [53–55] and neurological patients [48,49,52,56]. In contrast, studies using fMRI neurofeedback to train primary motor cortex (M1) upregulation reported inconsistent results: while some studies found no significant modulation [57,58], one study reported activation only for a subset of participants [59], and other studies even reported deactivation of M1 [54,60]. The neurophysiological basis for these inconsistent findings, particularly the lack of BOLD activation in M1 during motor imagery, has been a matter of debate [43,54].

To date, most fNIRS neurofeedback studies targeting the motor domain have focused on modulating SMA and PMC activity [3,10]. Although these regions are typically located beneath the hairy scalp, robust cognitive strategies (e.g. motor imagery) have been shown to elicit reliable fNIRS signals [61,62]. In addition, initial well-controlled clinical trials using fNIRS neurofeedback for stroke neurorehabilitation have shown promising results [63,64]. Furthermore, there is evidence that M1 may also be suitable for fNIRS neurofeedback interventions [65]. In a feasibility study, Matarasso *et al*. [66] combined the complementary strengths of fMRI and fNIRS neurofeedback: stroke patients first underwent three fMRI neurofeedback sessions to increase hand–knob activity during wrist extension training. FMRI then informed channel selection for the subsequent 10 fNIRS neurofeedback sessions in which patients also received neural-triggered functional electrical stimulation (FES) to additionally support the wrist movements. Thus, the use of fMRI enabled higher spatial specificity while fNIRS allowed for combining the neurofeedback training with FES and a large number of sessions. This interesting approach requires further validation investigating whether the fMRI–fNIRS combination actually outperforms the use of fNIRS alone.

### (b) Prefrontal brain function

Prefrontal brain regions are involved in a range of higher cognitive functions, including language production [67], regulation of affective processes [68], executive cognitive functions such as inhibitory control [69] and working memory [70]. Impairments in these functions and associated aberrant brain activity in prefrontal areas have been linked to several neurological and psychiatric diseases [71,72]. Neurofeedback represents a promising approach in this context to ‘normalize’ brain activity not only to treat diseases but also for neuroenhancement and age-related prevention. Note that prefrontal areas are particularly suitable for fNIRS measurements, as the absence of hair on the forehead usually results in good signal quality.

As an example, the inferior frontal gyrus (IFG) plays a crucial role in cognitive control and attention, which has implications for neurodevelopmental disorders such as attention-deficit/hyperactivity disorder (ADHD) [73]. In an fMRI neurofeedback study, the right IFG was trained in ADHD patients to alleviate these symptoms [74,75]. Moreover, a recent double-blind randomized controlled trial (RCT) reported significant improvement in clinical symptoms, however, effects were not superior to a sham control [75]. Initial clinical investigations for fNIRS neurofeedback in ADHD have been conducted in both adult and paediatric cohorts, but have yielded mixed findings so far [76–79].

In addition to the IFG, the middle frontal gyrus, particularly the dorsolateral prefrontal cortex (DLPFC) within it, is also essential for cognitive control. The DLPFC has been the focus of several fMRI neurofeedback proof-of-concept studies aimed at modulating its activity, particularly in the context of improving working memory [80–82]. Such protocols could provide a basis for future translation, for instance, in the context of cognitive decline (see for review, [83]). Recently, a proof-of-concept for fNIRS neurofeedback on working memory in healthy individuals was presented [84]. In the clinical context, a proof-of-concept study has shown that a short training protocol has the potential to mitigate cognitive decline in stroke patients [85]. Another proof-of-concept study, also focused on memory function, used an alternative approach. It used resting-state fMRI data and functional connectivity (FC) analysis, placing a seed in the hippocampus, to identify cortical connections suitable for targeting with fNIRS [86]. This approach resulted in the identification of a target in the temporal lobe. The study demonstrated the feasibility of this fMRI-informed approach and reported increased fMRI hippocampal activation following fNIRS neurofeedback, as well as improved scores in an associated memory task [86].

Other prefrontal functions in which the DLPFC is involved include appetite control and emotion regulation. A first RCT has demonstrated feasibility of DLPFC fMRI neurofeedback in obese individuals within a single training session [87]. An initial 12-session clinical trial of fNIRS neurofeedback of the DLPFC provided promising results in reducing binge-eating episodes in related disorders, improving secondary symptoms and executive functions, and thereby demonstrating the high potential of fNIRS for the clinical translation [88]. FMRI neurofeedback of the DLPFC has also shown positive results for emotion regulation in depressed patients, including one RCT [83,89]. However, larger RCTs are needed to provide evidence for the specificity and clinical relevance of symptom improvements [83]. FNIRS may be particularly suitable for achieving this goal, and first studies have provided proof-of-concept of DLPFC fNIRS neurofeedback [90,91]. Finally, an uncontrolled proof-of-concept study has shown promising preliminary results in reducing symptoms in social anxiety disorder using fNIRS neurofeedback [90].

### (c) Language network function

The left IFG and posterior superior temporal gyrus (pSTG), also known as Broca’s and Wernicke’s area, are key brain regions of the language network [67] and promising ROIs for fNIRS neurofeedback. Previous fMRI neurofeedback studies have focused on upregulation of activity in these ROIs to modulate language processing [92], for instance, with the specific aim of promoting recovery from post-stroke expressive aphasia [93], thus demonstrating the feasibility of self-regulation through mental or covert speech. Other fMRI neurofeedback studies have trained these areas in a psychiatric context and examined modulation of IFG and pSTG activity as a possible treatment for auditory verbal hallucinations in psychotic disorders such as schizophrenia [94,95]. The potential has been further highlighted in several reviews, also given the need to find new interventions aimed at modulating this highly treatment-resistant symptom [5,96–98]. However, the use of fMRI in this population may be limited as patients may have difficulty tolerating the MRI scanner environment. Moreover, schizophrenic patients sometimes have impaired learning abilities, which may require additional sessions for more effective neurofeedback, a challenge that fNIRS can address and that was examined in a case study [99].

### (d) Social network function

Lastly, first fMRI neurofeedback studies have begun to target higher social cognitive functions (see for review [100]). In this context, virtual human avatars provide an attractive way for more immersive feedback. For instance, a study in adolescents and young adults with autism spectrum disorder (ASD) targeted the FC between the somatosensory cortex and the superior temporal sulcus (STS), two areas associated with social cognition [101]. Without explicit feedback instructions, the experimental group learned to self-regulate the FC between the target ROIs, while a control group did not. The study reported successful self-regulation within four sessions as well as behavioural effects in the experimental, but not in the control group. Another early phase (IIa) RCT applying fMRI neurofeedback in young adults with ASD provided proof-of-concept for posterior STS upregulation over five sessions, in which participants were instructed to imagine (happy or sad) facial expressions while receiving feedback from a virtual avatar face [102]. Noteworthy, behavioural effects were also retained at a six-month follow-up assessment.

Regarding fNIRS neurofeedback to modulate higher cognitive social functions, a first randomized controlled proof-of-concept study has successfully targeted the right temporoparietal junction (rTPJ) in two groups of healthy individuals, either upregulating or downregulating the rTPJ using a virtual human avatar feedback display [103]. While the study demonstrated the feasibility of self-regulation in the upregulation group and behavioural effects related to spatial attention, no specific effects on social cognition were found [103]. Lastly, a controlled case study tested the feasibility of upregulating a temporal and prefrontal ROI in combination with the aim of improving face recognition capacities in ASD patients [104]. While one ASD patient received real feedback from these ROIs, the control patient received sham feedback from randomly generated brain signals. Successful upregulation of the ROIs as well as improved facial recognition capacities was reported only for the ASD patient who received real fNIRS neurofeedback. Given that involved target ROIs are well measurable with fNIRS and the high relevance of social cognitive processes in mental health [105], future larger fNIRS neurofeedback studies and RCTs are highly desirable.

## 3. Advancing fNIRS for neurofeedback applications: challenges and current solutions

**Figure 2.**
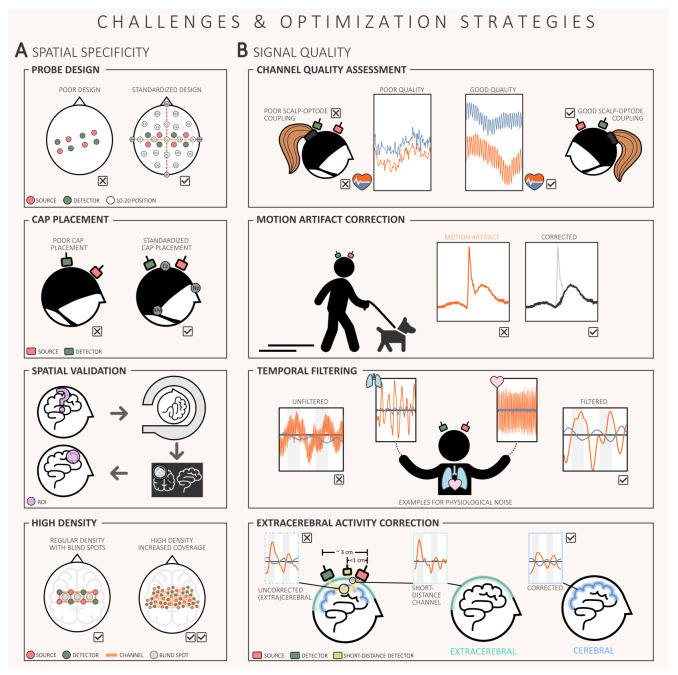
Illustration of possible optimization strategies to improve fNIRS neurofeedback applications with regard to the challenges of (*a*) spatial specificity and (*b*) signal quality. More specifically, part (*a*) shows various methods to improve spatial specificity, such as adequate probe design, precise cap placement, the possibility of spatial validation and the prospective application of high density measurements for more accurate neurofeedback. Part (*b*) focuses on improving signal quality and highlights important techniques such as channel quality assessment, motion artefact correction, temporal filtering and extracerebral activity correction to reduce artefacts and thus improve the reliability of fNIRS data.

As shown in table 1, there are several fNIRS-specific challenges that should be considered when planning and conducting fNIRS neurofeedback studies. So far, processing of fNIRS data and reporting results lacks a certain level of standardization [106,107], which also applies to real-time analysis of fNIRS data [108]. To ensure the effectiveness of fNIRS neurofeedback, a careful and informed selection of the methodology used is therefore required. Two important areas to consider in this context are improving spatial specificity (cf. figure 2*a*) and increasing signal quality (see figure 2*b*) [108]. Moreover, enhancing the overall research quality of a study is crucial to achieve generalizable and reproducible results [3]. In the following sections, we briefly discuss these three aspects and suggest concrete strategies that could help improve future fNIRS neurofeedback applications.

### (a) Improving spatial specificity

When training certain brain regions, it is important to aim for the greatest possible spatial specificity (i.e. the localization of brain activity) with the chosen brain-imaging technique [62,108,109]. The spatial specificity of fNIRS lies somewhere between the high spatial resolution of fMRI and the relatively low precision of EEG. However, it is important to note that fNIRS is only sensitive to activity within the superficial cortical layers of the brain [19,26,110]. Furthermore, an inherent limitation of fNIRS is the lack of individual anatomical information and limited head coverage due to the typically small number of available optodes [62,111–113]. Therefore, when planning and conducting a study, special attention should be paid to improving spatial specificity [108].

#### (i) Probe design and spatial validation

Particular attention should be given to the probe design (cf. figure 2*a*—probe design), that is the arrangement of source and detector optodes in the cap, to ensure accurate measurement of the target brain regions [108]. The probe allows effective targeting of the ROI and therefore can have a direct impact on the reliability, reproducibility and precision of the intervention itself [108,112–114]. To design probes based on either individual anatomy or standardized head models, specialized software is typically used [112–114]. Some of these software tools also provide simulation capabilities to evaluate how sensitive the probe is to targeting specific ROIs [112,115]. If higher accuracy is desired, prior fMRI validation (i.e., individual ROI localization; cf. figure 2*a*) can be performed, which in addition to higher spatial specificity, can also improve task sensitivity [62,109].

#### (ii) Cap placement

Precise placement of the fNIRS cap is another important aspect for improvement (cf. figure 2*a*—cap placement). Although the probe is designed to target a specific ROI, the lack of anatomical information coupled with the lack of a consistent and standardized placement method could affect the accuracy of precise targeting [108,116,117]. Since neurofeedback applications require repeated measurements on the same person, it is even more important to standardize cap placement so that it does not depend on the individual practical experience of the experimenter. Placement methods range from using standardized procedures (e.g. standardized EEG 10–20 positions to ensure, for instance, placement of Cz over the vertex using nasion, inion, left and right preauricular points) [108,118] towards more advanced techniques that require, for example, individual anatomy and task-dependent activation peaks [108,109,117]. While the latter approach offers greater individual accuracy, it is important to consider whether the benefits justify the additional time and cost in each individual case [109].

#### (iii) High density

Future fNIRS neurofeedback applications could benefit from currently emerging high-density measurements such as high-density diffuse optical tomography (HD-DOT) [29,31]. An important additional advantage here is that the sensitivity to the target ROI can be improved, as this helps to overcome the inherent blind spots [119] of standard regular density probe designs (cf. figure 2*a*—high density).

### (b) Increasing signal quality

Unlike offline processing, where the fully captured time series is typically analysed after data recording is complete, real-time processing analyses each new incoming data point immediately [108,120]. Accordingly, real-time processing is critical in systems such as brain–computer interfaces and neurofeedback implementations because both interact with real-time brain signals [121]. However, real-time processing presents some challenges, especially since not all (pre-)processing algorithms available offline can be easily applied in real time [108]. Furthermore, inaccuracies in real-time data processing cannot be corrected *post hoc*, as is possible during offline analysis. It is therefore particularly important that the real-time signal is of sufficient quality to ensure a certain level of precision and reliability. With regard to neurofeedback applications, this is particularly important as reduced signal quality can have a direct impact on the user experience and would result in the feedback being based on noise rather than representing meaningful brain activity [108,122].

To date, fNIRS research has mainly used continuous-wave fNIRS systems, which are widely used due to their ease of use, portability and affordability [18,26,31], making them also very suitable for real-time applications. Since these systems are unable to distinguish between different tissue layers or between absorption and scattering phenomena, they cannot be used to quantify absolute values, but rather concentration changes of oxygenated ( Δ[HbO] ) and deoxygenated ( Δ[HbR] ) haemoglobin. These changes are calculated using the modified Beer–Lambert law (mBLL) [18,26]. With offline analysis, the conversion of raw light intensity data into changes in optical density is based on a stable baseline obtained from the entire recorded time series. In contrast, real-time applications are often based on a shorter, initial baseline [108,120]. To achieve reasonably stable mBLL calculations in real time, it is recommended to record a sufficiently long and motion-free baseline (e.g. starting with at least 20−30 s baseline) before starting an experiment. However, since there is still no systematic validation in this regard, an exact recommendation of the optimal baseline duration is not yet possible [108,120].

A larger issue is the variety of noise sources typically included in the fNIRS signal [18,33,36]. An insufficiently cleaned fNIRS signal might result in neurofeedback being based on noise instead of brain activity [122]. Therefore, it is important to understand these factors and their potential impact on neurofeedback and to develop strategies to address these fNIRS-specific challenges in order to be also effective in less controlled, real-world environments [108].

#### (i) Channel quality assessment

The quality of the signal is often influenced by how well the optodes are (physically) coupled to the scalp, and there are various methods to assess data quality in this regard [108,123,124]. Among them, the scalp coupling index (SCI) is particularly suitable because it indicates the presence of the heartbeat in the fNIRS signal, which in turn reflects the strength of the optode–scalp coupling (cf. figure 2*b*—channel quality assessment) [123,124]. A high SCI value indicates good signal quality, and channels with SCI values that are too low often indicate poor quality channels that should be removed from subsequent analysis [108,123,124]. Real-time monitoring tools like PHOEBE provide a way to continuously assess signal quality by enabling real-time SCI tracking [124]. Although not yet common practice in real-time applications [3], these tools have the potential to immediately detect signal degradation and directly exclude poor quality data from affecting the overall analysis [108]. Initial approaches to monitoring signal quality in real time have also been adopted in commercial toolboxes for real-time data processing (Turbo-Satori, Brain Innovation B.V., Maastricht [120]).

#### (ii) Motion artefact correction

Motion artefacts, often caused by (physical) optode shifts (e.g. due to head movement), can cause signal spikes and baseline shifts and seriously affect data quality (cf. figure 2*b*—motion artefact correction) [125,126], which could negatively impact the accuracy of the neurofeedback information provided [108]. Although there are various methods for correcting motion artefacts for fNIRS [125–130], their real-time application is not always possible. As a result, this crucial corrective step is often missing in many neurofeedback studies [3]. Additionally, fully automated offline motion correction methods such as the temporal derivative distribution repair (TDDR) method [128] hold potential for adaptation to real-time analysis. However, they have not yet been fully validated for this purpose [108].

#### (iii) Temporal filtering

While the presence of the heartbeat is an important indicator of the quality of the fNIRS signal [124], it is also classified, along with respiration, Mayer waves and other low-frequency oscillations, as non-evoked cerebral and extracerebral systemic activity—or simply as physiological noise [18,33]. However, the frequency bands of some of these noise components are often distinguishable from the task frequency, allowing the use of temporal filters to separate them [131] (cf. figure 2*b*—temporal filtering). Temporal filters are also often used in real-time preprocessing [3]. However, it is important to carefully adjust and test filter settings to ensure that they reduce physiological noise without affecting the task frequency. Before applying filters, information about the potential challenges and filter options should be obtained. There are already some resources for both offline and real-time fNIRS analysis (e.g. [108,131]).

#### (iv) Extracerebral activity correction

In addition to the previously mentioned physiological noise, task-evoked cerebral and extracerebral systemic activity forms another component of noise in the fNIRS signal [18,33]. Correcting these artefacts is significantly more difficult due to possible overlap with task frequencies, making conventional temporal filters insufficient [18,33,36,122,132–134]. However, if this noise component remains uncorrected, data interpretation becomes complicated because this component can either mimic or mask actual brain signals [33]. Direct correction of task-evoked cerebral components is not currently possible, but for task-evoked (and non-evoked) extracerebral components, the gold-standard for correction involves the use of short-distance channels (SDCs) [108,122,132,133]. SDCs have a source–detector distance of less than 1 cm (ideally 0.8 cm for adults [135]), which allows them to primarily detect extracerebral signals (cf. figure 2*b*—extracerebral activity correction). The data can then be used to remove extracerebral influences from the normal fNIRS channels, which is often done using regression-based approaches [18,33,108,122,132,133,136,137]. Although these methods are generally suitable for real-time applications, they have not yet been widely used in neurofeedback studies [3]. However, in cases where SDCs are not available, alternative correction methods have been proposed [25,108,122,133,134] and first approaches have already been implemented in neurofeedback pipelines [3].

### (c) Enhancing research quality

Although the issue of research quality is not limited to fNIRS research, Kohl *et al*. [3] found that the design and reporting quality of fNIRS neurofeedback studies are predominantly moderate, ranging from low-quality studies to studies that used robust methods such as sham control conditions, randomization and blinding. A common factor was small sample sizes which reduce statistical power and thereby the ability to detect true effects. Nevertheless, several strategies are available to enhance the quality of research in this area [3].

#### (i) Clear definition of study type

It may sound trivial, but properly categorizing a neurofeedback study (e.g. as feasibility, pilot or proof-of-concept study) and appropriately reporting it in the publication are important. It is equally important to explicitly state whether the analysis is planned (i.e. based on preformulated hypotheses) or exploratory. This clarity in setting objectives, design and analysis steps can significantly improve the accuracy of reporting and prevent the risk of excessive conclusions [3,107].

#### (ii) Sampling plan

It is important to have transparent sampling plans to ensure the validity and precision of the research [3,107]. This includes carefully considering the number of planned neurofeedback sessions and the participants required (i.e. sample size) to detect a specific effect of interest on the main outcome measure(s). In addition to sample size planning, the number of repeated measures should also be considered, especially given the high scalability of fNIRS, which makes it ideal for multisession interventions. While *a priori* power or sensitivity analyses are commonly used when planning sample sizes, it is important to consider potential biases caused by resource limitations such as limited funding or time constraints [107]. These limitations may result in smaller sample sizes and potentially biased effect sizes. Ensuring an adequate sample size is thus crucial to effective research. Planning sample sizes based on realistic effect sizes from literature that is less susceptible to publication bias (e.g. Registered Reports) [138] or the use of established smallest effect sizes of interest can significantly improve the overall integrity, evidential strength and trustworthiness of the results [139]. This is particularly important for non-significant results [140]. To increase the chance of drawing meaningful and reproducible conclusions, researchers should avoid basing power analyses on (typically small sized) pilot studies [141]. If effect-size estimates cannot be well justified, other sample size planning strategies may be more appropriate [142]. Regardless of the methodological approaches (e.g., frequentist or Bayesian), the assumptions underlying sample size decisions should always be transparent and justified [3,107,142].

In neurofeedback research, which often involves a repeated-measures design, it is important to include this aspect in sample size calculations, particularly in studies that focus on primary outcome variables tested with a repeated-measures test [3]. However, we further note that caution should be exercised when using common software such as GPower for power or sensitivity calculations in repeated-measures ANOVA [143]. Some studies [144,145] have pointed out that the default effect size option in GPower can lead to inaccurately low sample sizes and sensitivity measures. To prevent underpowered/insensitive studies, the effect-size setting should be adjusted based on whether a purely within- [144] or between-group design with interaction [145] is used. For a more detailed overview of effect-size derivations, see also Kieslich [146].

#### (iii) Control conditions

Another key aspect in neurofeedback research is the selection of appropriate control conditions [3,38,147]. For an accurate assessment of the effectiveness of neurofeedback, it is important to include a control condition (or group), for instance, treatment as usual, sham feedback, bidirectional regulatory control and/or randomized ROI control [38]. Incorporating such controls ensures that the observed results are actually based on the feedback and not on other non-specific factors. Ideally, and if resources allow, the integration of multiple control conditions may enable a clearer distinction between neurofeedback-specific effects and general, non-specific processes [3,38,39].

#### (iv) Bias reduction

In neurofeedback research, biases can influence results [3]. A selection bias can arise, for instance, from non-random assignments. This problem can be solved using randomization and hidden assignments [148]. Furthermore, the expectations of the participants or the experimenter could also influence the neural responses. Strategically assigning participants to different feedback conditions and ensuring that experimenters are blind to these conditions can mitigate such expectancy effects [3]. When blinding is not possible, the use of standardized scripts or automation can be an effective alternative. Moreover, comprehensive blinding can counteract performance and detection biases arising from unmasked participants or inconsistent outcome measures [148]. Effective management of drop-outs is critical to minimizing attrition errors. Additionally, the use of open science practices such as preregistration helps reduce selective reporting and reporting errors [107,148–151].

## 4. Perspectives of fNIRS neurofeedback: an outlook

FNIRS neurofeedback is a rapidly developing field with numerous promising perspectives for future development. Further advances in hardware and methodology could help expand the application of neurofeedback to real-world environments. Additionally, making the hardware and software more user-friendly could expand usage to a wider range of users and scenarios. In this discussion, we share our ideas for innovative concepts and identify areas where we expect major progress towards these goals. In addition, we explore some exciting possible applications and discuss possible requirements that are crucial to realizing these visions.

### (a) Advancements in hardware and methodology

#### (i) Multimodal neurofeedback

It is likely that no single functional neuroimaging tool is perfect for all neurofeedback applications or equally effective for every person and situation. Different signals such as electrophysiological and haemodynamic signals might have different effects depending on the scenario or person. Accordingly, a combination of EEG and fNIRS could help provide a more comprehensive and accurate view of brain activity [152,153]. Such a multimodal approach, already being considered in the field of brain–computer interfaces [154], combines the high temporal resolution of EEG and the spatial specificity of fNIRS and could potentially improve the effectiveness and efficiency of neurofeedback [155]. Because both EEG and fNIRS are mobile technologies, this multimodal approach is well suited for use in real-world environments and paves the way for more effective and personalized neurofeedback protocols that could benefit a wider range of users.

#### (ii) High-density (multivariate) neurofeedback

In addition to univariate ROI-based methods, new approaches such as FC and multivariate approaches [41,156] promise a possible further development of neurofeedback. There are already initial approaches to FC-based neurofeedback in the fMRI field [157]. Thus, the application of fNIRS neurofeedback for FC training in diverse networks could examine a broader range of functions. Despite the reliance on correlational analysis, well-designed FC-based neurofeedback has the potential to advance FC research and facilitate the testing of causal hypotheses [158,159]. Compared to low-density fNIRS, the use of HD-DOT systems offers potential for fNIRS neurofeedback as it enables higher spatial specificity and depth resolution [29,160], which could result in more precise targeting of ROI(s) and reduce typical blind spots compared to standard probe designs [119] (cf. figure 2*a*—high density). Since the first commercial wearable HD-DOT systems are already available [29–31], these devices would also be suitable for wearable neurofeedback, although this has not yet been demonstrated. The higher spatial resolution offered by HD-DOT and other high-density fNIRS systems could enable better detection of more complex brain activation patterns, which could lead to the development of more personalized neurofeedback. The integration of HD-DOT with approaches such as FC and multivariate techniques therefore provides an interesting opportunity to develop more advanced fNIRS neurofeedback protocols.

#### (iii) Smartphone-based, artificial intelligence-supported wearable neurofeedback

While significant progress has been made in the mobility and portability of fNIRS devices [30,153,161–163], the future of fNIRS neurofeedback in real-world environments may benefit from further hardware and methodological developments. In contrast to the idea of high-density fNIRS neurofeedback, an important step would be to further miniaturize fNIRS devices into smaller, more comfortable wearables that are easy to use and allow individuals to use them independently of experts. To better integrate neurofeedback into everyday life, mobile applications (apps) should be developed that synchronize with these wearable fNIRS systems and provide real-time neurofeedback visualization as well as data collection and processing capabilities. There have already been technological developments in the EEG area, such as significantly smaller devices [164–167] and app-based real-time processing [168–170]. A key advantage of EEG is that, due to its lower spatial specificity, it allows activity to be recorded using devices positioned nearby or in the ear [164–167]. FNIRS, on the other hand, requires more precise placement to capture the activity of specific ROIs. Accordingly, the development of small fNIRS wearables additionally requires methods for innovative positioning mechanisms that could, for example, use artificial intelligence (AI) for precise placement. AI could also personalize (app-based) neurofeedback protocols by identifying and training individual brain patterns (e.g. [171], for a review about AI in EEG-based BCI).

In addition, wearable fNIRS neurofeedback in telemedicine or telerehabilitation are another exciting prospect, where there are already initial approaches in the EEG field [172,173]. Integration with (mental) health apps and wearables such as smartwatches and the associated tracking of various health metrics enables improved monitoring of a person’s well-being [155]. These data could further individualize fNIRS neurofeedback and tailor sessions based on daily activities or physiological states such as physical activity, sleep patterns and physiological information (e.g.heart rate). In addition, physiological wearable data could improve (real-time) fNIRS signal quality and help with artefact correction [36,108,137,174]. In addition, various environmental factors such as lighting, ambient noise and temperature can potentially affect the performance of wearable neurofeedback. This information could be measured and used to instruct users to change their environment when necessary, thus improving neurofeedback sessions in various real-world environments. However, to develop smartphone-based and AI-powered wearable neurofeedback, important privacy and security issues should be addressed [175,176]. Because these devices process sensitive health information, strict security measures are essential to protect against unauthorized access and misuse of these data. Therefore, when developing such apps, the balance between the benefits of AI and smartphones and protecting the privacy of personal data should always be kept in mind.

### (b) Future applications

#### (i) Using fMRI for validating fNIRS neurofeedback

In a more research-oriented context, the combined (simultaneous and/or sequential) use of fMRI and fNIRS represents a promising opportunity to better validate and improve fNIRS neurofeedback [20,62]. For example, fMRI could serve as a pre- and post-measurement tool for fNIRS neurofeedback to examine changes in brain function before and after neurofeedback intervention. Furthermore, these data could improve the spatial specificity of fNIRS through spatial validations [62] (cf. §3a and figure 2*a*—spatial validation), provide insights into immediate effects of neurofeedback (via fNIRS), and track long-term changes in brain activity (via fMRI). The combination of fNIRS and fMRI could thus provide a better overview of how neurofeedback affects brain activity across sessions and in different brain regions, which could be used to refine neurofeedback protocols, better understand their mechanisms and identify areas for future research. However, such approaches are associated with considerable costs, as fMRI measurements are expensive and fMRI-compatible fNIRS devices must be purchased. These costs should therefore be taken into account in advance, for instance, when applying for funding.

#### (ii) Fingerprinted neurofeedback

Creating so-called ‘fingerprints’ to identify individuals is typically based on brain activity patterns derived from a single functional neuroimaging method [177]. However, this approach can also be used to create individual fingerprints based on higher spatial resolution methods such as fMRI, increasing the reach of methods with lower spatial specificity (e.g. EEG or fNIRS) to deeper brain regions [178]. In the EEG field, for instance, this approach has been used to create a type of filter for fingerprinted EEG neurofeedback based on previously recorded simultaneous fMRI-EEG data to train brain areas such as the amygdala [179–182]. Due to the conceptual similarity between fNIRS and fMRI, this approach is particularly promising for fNIRS neurofeedback. However, the idea of creating fingerprints based on fMRI data [22,177,183] and then using them in fingerprinted fNIRS neurofeedback has not yet been implemented. The use of machine learning or AI-based methods could be particularly effective in this context. These technologies allow for more precise analysis of simultaneously acquired data and could potentially find more subtle patterns, improving the effectiveness and individualization of neurofeedback training.

#### (iii) FNIRS hyperfeedback

In hyperscanning, multiple individuals are measured simultaneously which can offer interesting insights into phenomena such as inter-brain synchrony [184]. An important aspect of inter-brain synchrony is that brain activity of two or more persons become synchronized during interaction, which is considered crucial for various cooperative behaviours or effective communication [184]. Hyperscanning neurofeedback (or ‘hyperfeedback’), provides feedback based on a common target parameter resulting from the brain activity of all involved individuals and thus requires the cooperation of these subjects to regulate this parameter together. This could in turn lead to an increase in empathy or social belonging [184]. Although this field is still in its infancy, several studies on EEG hyperfeedback have already been conducted [185–189]. However, research on haemodynamic hyperfeedback is still limited [184,190,191]. Given that social interactions likely work best in face-to-face, real-world environments, the potential for using fNIRS in this context is particularly promising. This application represents an interesting direction for future research, but more and sufficiently large studies are needed in the future to determine its effectiveness [184].

### (c) Essential foundations for future progress

#### (i) Analysis and reporting of change in both oxygenated and deoxygenated haemoglobin Δ[HbR]

In previous fNIRS-guided neurofeedback studies, Δ[HbO] was predominantly used as a feedback signal [3]. This problem is not necessarily specific to fNIRS neurofeedback research, but is strikingly common in the fNIRS field in general [106,192,193]. This choice is often justified by the higher amplitude of Δ[HbO] which can more easily produce significant results compared to Δ[HbR] [193], or simply not justified at all. Although it may be useful to select a single signal type for neurofeedback, *post hoc* data analysis and results reporting should not be limited to only the selected signal and completely ignore the other signal. Furthermore, basing this preference on amplitude difference justification alone is not particularly convincing, since the suitability of Δ[HbO] or Δ[HbR] can potentially vary from person to person and also between different brain regions and specific tasks [122,194]. Future fNIRS studies should therefore better justify the chromophore choice for their neurofeedback protocol, for instance, on the basis of protocol-specific pilot measurements or validation studies. In line with previous recommendations [106,107], both Δ[HbO] and Δ[HbR] results should always be reported, regardless of the chromophore choice for neurofeedback, to enable a better understanding of the underlying brain activity and thereby improve the further development of neurofeedback interventions.

#### (ii) Promoting open science practices

The advancement of the neurofeedback field and most of the ideas discussed depends, among other things, on a common factor: the transparent and open exchange of data and analysis codes. Especially when it comes to AI applications and machine learning algorithms, access to large datasets is required to train and validate models and to reduce bias [195,196]. It is likely that AI will play a larger role in real-time applications in the future [197], so the availability of diverse and large datasets will be crucial for developing AI models. In addition, the open sharing of data and code enables the validation of results and the replication of studies, which can not only promote collaboration in the scientific community but also accelerate innovation in the field [107,198–201]. To share data efficiently, a certain level of standardization should be maintained and the FAIR principle should be followed (i.e. findable, accessible, interoperable and reusable) [107,198–200]. Inter-operability and reusability of fNIRS can be achieved when data are stored in the shared near-infrared spectroscopy format (.snirf) [202], an open access data format developed by the fNIRS community [107], and shared within the Brain Imaging Data Structure [203] extended for fNIRS [204], which sets a standard for organizing and naming data and metadata [107]. To make the data accessible and findable, various platforms and repositories can be used (for an fNIRS-specific example, see https://openfnirs.org/data/ [107]).

In addition, preregistration before starting the study is an important aspect to increase the credibility of fNIRS (neurofeedback) research. This includes publicly describing the research methodology, including hypotheses, experimental procedures and data analysis plans [107,205–208]. Preregistration helps prevent selective reporting and ensures that research is guided by *a priori* planned hypotheses and methods [107]. Furthermore, journals (and community platforms) are increasingly offering peer-review for preregistrations in the form of so-called Registered Reports (RRs) [138]. After a positive peer-review based on the study protocol, authors receive a so-called in-principle acceptance as a guarantee for the publication of their final study if it complies with the approved protocol. As a result, RRs allow publications independent of statistical outcomes and are therefore a promising way to mitigate publication bias (i.e. preferred publication of significant study outcomes) as indicated by first meta-analyses [149,150].

Finally, it is important to carefully document every aspect of a study, from methodology to inconsistencies and even deviations from the original plan, to maintain scientific integrity [39,107]. Therefore, researchers can adhere to established guidelines when reporting their studies [106]. This also includes standardizing results reporting and using consistent metrics and statistical methods to present results to make it easier to compare the results of different studies. A current initiative in the fNIRS area pursues this goal in particular (FRESH study: https://openfnirs.org/data/fresh/). Standardization is also important in meta-analyses and systematic reviews, which are crucial for synthesizing evidence and drawing broader conclusions in the field.

## 5. Conclusion

FNIRS allows targeting brain regions with a sufficiently high spatial specificity at low cost and is therefore particularly suitable for neurofeedback applications. Although fNIRS presents its own challenges, it remains the only technique that can effectively integrate haemodynamic-based neurofeedback into real-world environments such as bedside, home use and on-the-go mobile scenarios. Accordingly, fNIRS offers many exciting possibilities for haemodynamic-based neurofeedback applications and, due to its compatibility with challenging populations such as children, combined with its applicability in real-world settings, enables unique options such as haemodynamic hyperfeedback to promote personal social interactions [184].

Although the future of fNIRS neurofeedback is full of potential and promising applications, there is a need for further development in this area. Accordingly, it is essential that fNIRS devices, software and analysis tools are further developed and open science practices are followed. We look forward to a future full of innovative research and applications such as haemodynamic hyperfeedback, multimodal neurofeedback, integration of AI algorithms and smartphone-based home training.

## Data Availability

This article has no additional data.
